# The Ablation Performance of Silicon Nitride/Boron Nitride Fibrous Monolithic Ceramics under an Oxyacetylene Combustion Torch

**DOI:** 10.3390/ma16206703

**Published:** 2023-10-16

**Authors:** Qingqing Chen, Yuan Zhang, Yu Zhou, Daxin Li, Guobing Ying

**Affiliations:** 1 School of Electronic Engineering and Intelligent Manufacturing, Anqing Normal University, Anqing 246133, China; 2 Department of Materials Science and Engineering, College of Mechanics and Materials, Hohai University, Nanjing 211100, China; zhangyuan795@163.com; 3 Institute for Advanced Ceramics, School of Materials Science and Engineering, Harbin Institute of Technology, Harbin 150001, China; zhouyu@hit.edu.cn (Y.Z.); lidaxin@hit.edu.cn (D.L.); 4 Key Laboratory of Advanced Structural-Functional Integration Materials and Green Manufacturing Technology, Harbin Institute of Technology, Harbin 150001, China; 5 School of Materials Science and Engineering, Harbin Institute of Technology (Shenzhen), Shenzhen 518055, China

**Keywords:** Si_3_N_4_/BN fibrous monolithic ceramics, mechanical, ablation resistance, oxyacetylene

## Abstract

In this study, Si_3_N_4_/BN fibrous monolithic ceramics were successfully prepared by wet spinning extrusion and hot pressing, and the effects on its ablation performance and microstructure were studied. The samples were burned in an oxyacetylene flame for 60 s × 30 to evaluate the ablation resistance. With the increase in ablation time, the fibrous monolithic ceramics exhibited specific mass and linear ablation rates, which show a trend of first increasing, then decreasing, and then increasing again. When the ablation time is 60 s × 10, 60 s × 20, and 60 s × 30, the mass ablation rates of the fibrous monolithic ceramics are 1 × 10^−5^ mg/s, −8.3 × 10^−6^ mg/s, −6.7 × 10^−7^ mg/s, respectively; the linear ablation rates are 4.7 × 10^−5^ μg/s, −1.2 × 10^−5^ μg/s and 1.7 × 10^−6^ μg/s. After 60 s × 30 of ablation, the surface oxides of the species are washed away by the oxyacetylene flame, revealing a porous coral-like structure with many cracks. A glass phase layer, predominantly constituted by sintering aids, envelops the Si_3_N_4_ ceramic surface on the ablated sample, serving as an effective barrier against additional ablation.

## 1. Introduction

Materials used in hypersonic vehicles’ radomes and antenna windows must withstand ultra-high temperatures, severe ablation, and intense thermal shocks during re-entry, all while transmitting electromagnetic signals [1,2,3,4]. Currently, only a select few nitride materials meet these stringent criteria. Si${}_{3}$N${}_{4}$, characterized by its strong covalent and atomic bonds, stands out as a top-performing structural ceramic. This material boasts superior mechanical properties, impressive thermal stability (decomposing at temperatures up to 1900 °C), and a relatively low dielectric constant (ranging from 4.9 to 7.9) coupled with minimal dielectric loss [5,6,7,8,9]. Silicon nitride, when employed as a radome material, merges the robustness and rain ablation resistance of alumina ceramics with the low dielectric constant and impact resilience of quartz ceramics. This unique combination has garnered significant attention in the materials science community. However, a notable drawback is that silicides tend to vaporize during ablation, leading to a coarse ablated surface [10,11,12,13,14,15,16]. In comparison, BN ceramics offer enhanced thermal stability and reduced dielectric constants and losses than Si${}_{3}$N${}_{4}$ ceramics. Boron nitride ceramics, which decompose at a staggering 3000 °C, surpass Si${}_{3}$N${}_{4}$ in terms of thermal stability, dielectric properties, and consistent thermal and electrical performance. Furthermore, Si${}_{3}$N${}_{4}$/BN composite ceramics, enhanced with BN, exhibit more consistent thermophysical properties, a reduced dielectric constant, and better machinability than their pure Si${}_{3}$N${}_{4}$ counterparts [17,18,19]. A caveat to consider is that BN, when exposed to oxidizing atmospheres exceeding 1000 °C, reacts with oxygen, producing B${}_{2}$O${}_{3}$ in either liquid or gaseous form. This oxidation process detrimentally impacts the mechanical properties of the material by forming oxide scales on its surface [12,20].

Pacquet achieved the synthesis of Si${}_{3}$N${}_{4}$-BN-SiO${}_{2}$ materials, exhibiting notable transparency and resistance to ablation at temperatures surpassing 2000 °C. Although these ceramics serve proficiently as antenna windows, their vulnerability to defects might result in sudden failures during intense thermal events [21]. Zou formulated Si${}_{3}$N${}_{4}$/BN composite materials employing the Precursor Impregnation and Pyrolysis method, with liquid boron nitride as the foundational precursor. The significant ablation observed in the Si${}_{3}$N${}_{4}$/BN composites is primarily due to the reactive oxidation of the embedded fibers and a limited availability of silicon-enriched glass [22]. Wang successfully produced a Si${}_{3}$N${}_{4}$/BN radome characterized by enhanced high-temperature ablative attributes through selective laser sintering. The data suggests that the application of cold isostatic pressing augments the densification of the Si${}_{3}$N${}_{4}$-Si${}_{2}$N${}_{2}$O-BN ceramics, thereby amplifying their resistance to high-temperature ablation [23].

The fibrous monomer Si${}_{3}$N${}_{4}$/BN, which consists of strong Si${}_{3}$N${}_{4}$ cells around a hexagonal arrangement of weak BN cell interfaces, has been found to exhibit high intensity non-catastrophic failure at both room and high temperatures due to the extensive interaction of cracks through the BN cell interface (crack lamination and crack deflection). Because this material is designed for use at high temperatures, the ablative behavior is one of the more important criteria that needs to be clearly understood before it can be applied.

The Si${}_{3}$N${}_{4}$/BN fibrous monolithic ceramics sintered under 1800 °C/20 MPa/2 h were subjected to ablation tests with an oxygen-acetylene flame for 60 s × 10, 60 s × 20, and 60 s × 30, respectively. The study investigated the influence of ablation time on the ablation resistance of Si${}_{3}$N${}_{4}$/BN fibrous monolithic ceramics and analyzed the ablation mechanism of this material system through phase, composition, and microscopic structural analysis of the ablated surfaces.

## 2. Materials and Methods

### 2.1. Raw Materials

**Figure 1:**
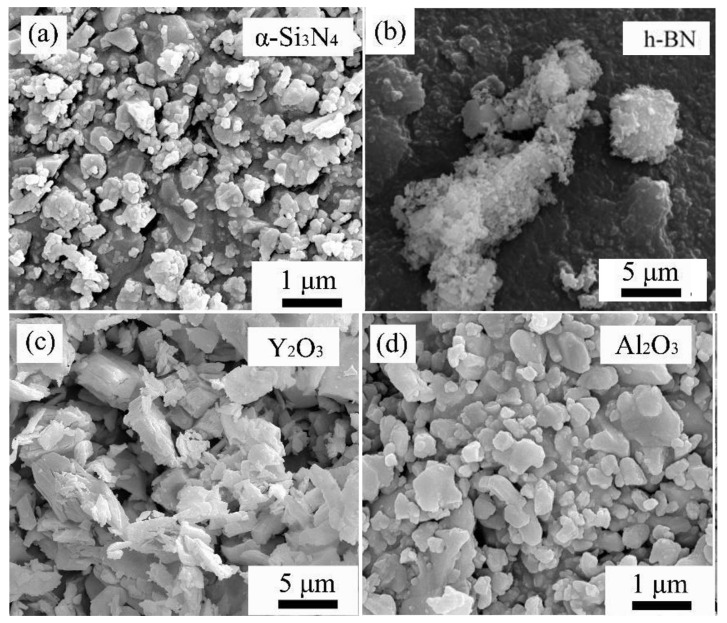
SEM morphologies of the raw powders: (**a**) α-Si${}_{3}$N${}_{4}$. (**b**) BN. (**c**) Y${}_{2}$O${}_{3}$. (**d**) Al${}_{2}$O${}_{3}$.

**Figure 2:**
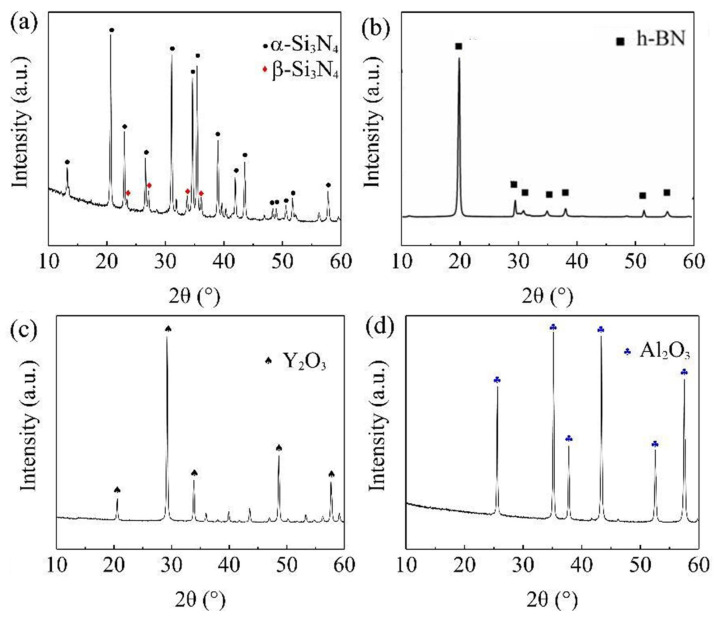
XRD patterns of the raw powders: (**a**) α-Si${}_{3}$N${}_{4}$. (**b**) BN. (**c**) Y${}_{2}$O${}_{3}$. (**d**) Al${}_{2}$O${}_{3}$.

The powders used in this experiment are α-Si${}_{3}$N${}_{4}$, BN, Y${}_{2}$O${}_{3}$ and Al${}_{2}$O${}_{3}$. The purity, particle size, and manufacturers of the raw materials are shown in Table 1. Figure 1 and Figure 2 are the SEM images and XRD patterns of the raw material powders, respectively. From the surface morphology, it can be observed that the raw material size distribution is uniform, and the size meets the requirements. From the X-ray diffraction spectrum, it is evident that the purchased α-Si${}_{3}$N${}_{4}$ and BN powders have high purity. The Si${}_{3}$N${}_{4}$ powder contains both α-Si${}_{3}$N${}_{4}$ and β-Si${}_{3}$N${}_{4}$ phases, with the α phase being dominant and a minor amount of the β phase.

### 2.2. Experimental Procedure

**Figure 3:**
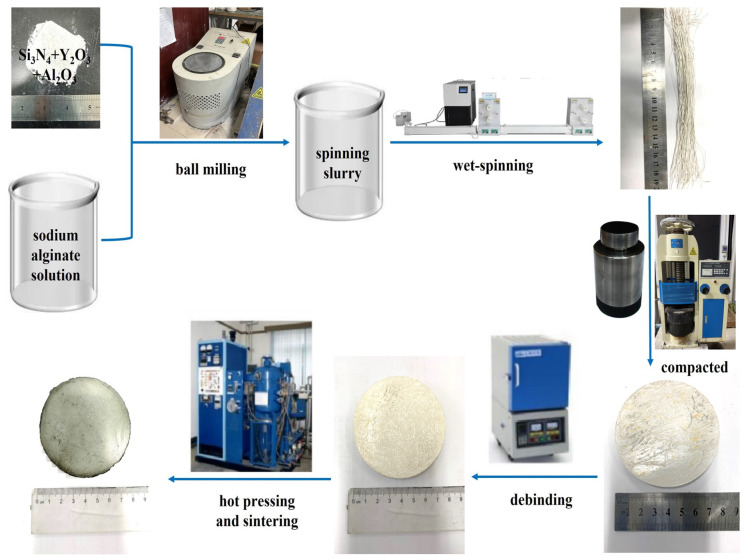
The process flowchart of Si${}_{3}$N${}_{4}$/BN fibrous monolithic ceramics.

The preparation method for Si${}_{3}$N${}_{4}$/BN fibrous monolithic ceramic is illustrated in Figure 3. Sodium alginate solution is mixed uniformly with the initial powder to obtain a spinning slurry, BN powder is dispersed in deionized water to obtain an interfacial layer solution. Table 2 shows the specific ratios. Si${}_{3}$N${}_{4}$ fibers are then extruded using a wet-spinning device and coated with BN. After air drying at room temperature, the fibers are trimmed to a specified size, aligned in a chromium steel mold, and pre-pressed at 20 MPa for 5 min, yielding Si${}_{3}$N${}_{4}$/BN fibrous monolithic ceramic green bodies. The green bodies are dried at 60 °C. Then, the samples were subjected to a controlled heating regimen in a muffle furnace, increasing at a rate of 1 °C/min until reaching 600 °C, where they were maintained for a duration of 1 h to facilitate debinding. Under a 0.1 MPa N${}_{2}$ atmosphere and a uniaxial pressure of 20 MPa, the equipment is heated at 15 °C/min to 1800 °C. After maintaining this temperature for 2 h, when the furnace cools to room temperature, the Si${}_{3}$N${}_{4}$/BN fibrous monolithic ceramics is removed from the mold.

### 2.3. Characterization

Bulk samples was conducted using X-ray diffraction (XRD). The equipment utilized for the XRD test was the D/max-RB type X-ray diffractometer from Rigaku Corporation.The microstructure of the material is characterized by imaging with a scanning electron microscope (SEM, including XL 30, TM 3000, HITACHI S4800, and Sirion 2000) and is analyzed using an energy dispersive spectrometer (EDS) to examine the micro-structure of Si${}_{3}$N${}_{4}$/BN fibrous monolithic ceramics.

Material ablation tests adhered to the GJB323A-96 standard [24], designed for materials such as heat shields, insulations, coatings, C/C composites, refractory metals, and high-temperature ceramics. The samples used in these tests measured 30 mm in diameter and maintained a thickness of 10 ± 0.1 mm. For this particular test, a 10 mm thick sample was employed. To ensure a surface roughness below 0.1 mm, the sample was meticulously ground. Throughout the ablation process, the oxyacetylene flame met the established standard and maintained stability. The sample underwent 60 s × 30 of ablation. The oxyacetylene ablation test apparatus, operated via a single-chip computer, relied on a turntable rotation to stabilize the flame pre-ablation and retract it post-ablation efficiently. Oxygen and acetylene, indispensable for the process, were sourced adhering to the GJB323A-96 standards for industrial gaseous oxygen and dissolved acetylene, respectively. The sourced oxygen and acetylene comprised no less than 99.2% and 98% purity, respectively. By contrasting sample metrics—weight and thickness, both pre and post-ablation, as well as the flame’s exposure duration—the mass and linear ablation rates were determined. Table 3 details the working parameters of the oxyacetylene flame.

Herein, defined in Formula (1), the Si${}_{3}$N${}_{4}$/BN fibrous monolithic ceramic’s mass ablation rate was derived:

(1) $$R_{m}=\left(m_{1}-m_{2}\right)/t),$$

where $R_{m}$ represents the sample’s mass ablation rate (g/s); $m_{1}$ stands for the original sample mass (g); $m_{2}$ indicates the post-ablation sample mass (g); and *t* is the ablation duration (s).

Formula (2) was employed to determine the Si${}_{3}$N${}_{4}$/BN fiber monolithic ceramic’s linear ablation rate:

(2) $$R_{d}=\left(d_{1}-d_{2}\right)/t,$$

where $R_{d}$ signifies the sample’s linear ablation rate (mm/s); $d_{1}$ represents its original thickness (mm); $d_{2}$ is its post-ablation thickness (mm); and *t* is the ablation duration (s).

## 3. Results and Discussions

### 3.1. Ablation Resistance

#### 3.1.1. Ablation Rate

**Figure 4:**
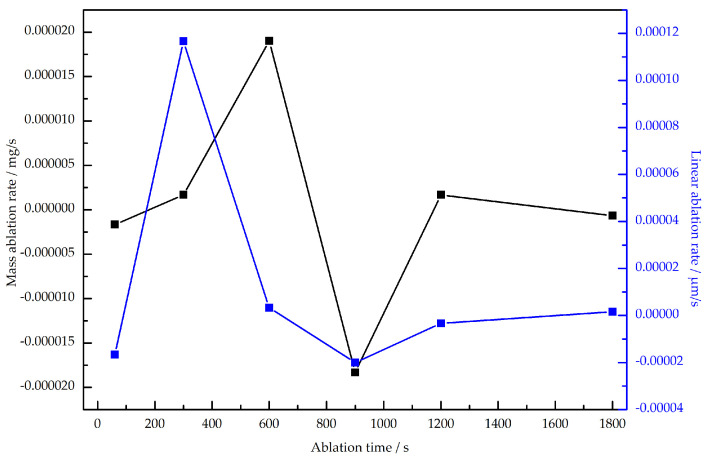
Ablation rate of Si${}_{3}$N${}_{4}$/BN fibrous monolithic ceramics.

The ablation resistance of Si${}_{3}$N${}_{4}$/BN fibrous monolithic ceramics is assessed through the linear ablation rate and mass ablation rate across various ablation durations, as demonstrated in Figure 4. It can be observed that with the prolongation of ablation time, the fibrous monolithic ceramics exhibited mass and linear ablation rates a trend of increasing first, subsequently decreasing, and then increasing again. Notably, during a specific phase, the ablation rate is negative, indicating that the fibrous monolithic ceramics primarily predominantly oxidation at this stage, resulting in an increase in material weight. For ablation times of 60 s × 10, 60 s × 20, and 60 s × 30, the corresponding mass ablation rates of the fibrous monolithic ceramics are $1\times 10^{-5}$ mg/s, $-8.3\times 10^{-6}$ mg/s, and $-6.7\times 10^{-7}$ mg/s, respectively; the linear ablation rates are $4.7\times 10^{-5}$μm/s, $-1.2\times 10^{-5}$μm/s, and $1.7\times 10^{-6}$μm/s. Table 4 reveals that the ablation resistance of Si${}_{3}$N${}_{4}$/BN fibrous monolithic ceramics is notably superior to other Si${}_{3}$N${}_{4}$-based ceramics.

#### 3.1.2. Macroscopic Morphology

**Figure 5:**
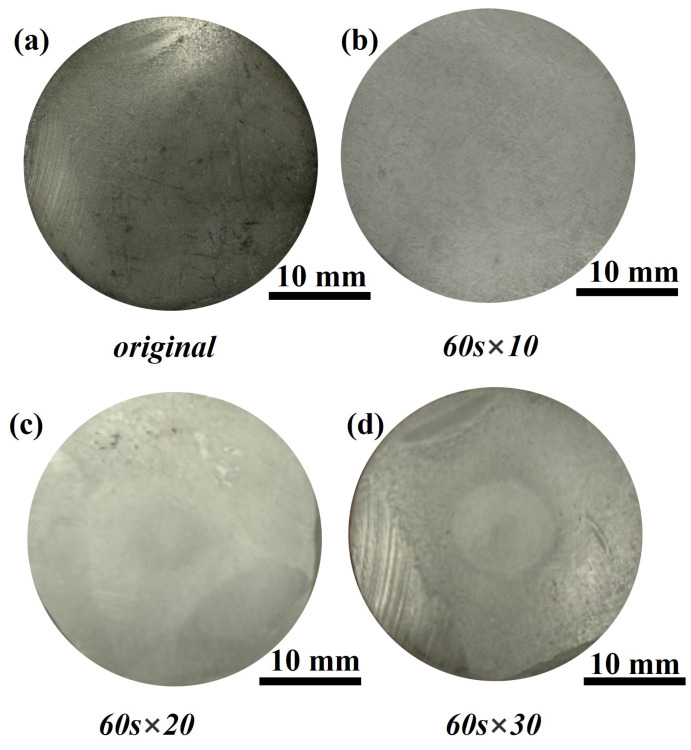
Macroscopic morphology of Si${}_{3}$N${}_{4}$/BN fibrous monolithic ceramics after different ablation time. (**a**) original. (**b**) 60 s × 10. (**c**) 60 s × 20. (**d**) 60 s × 30.

**Figure 6:**
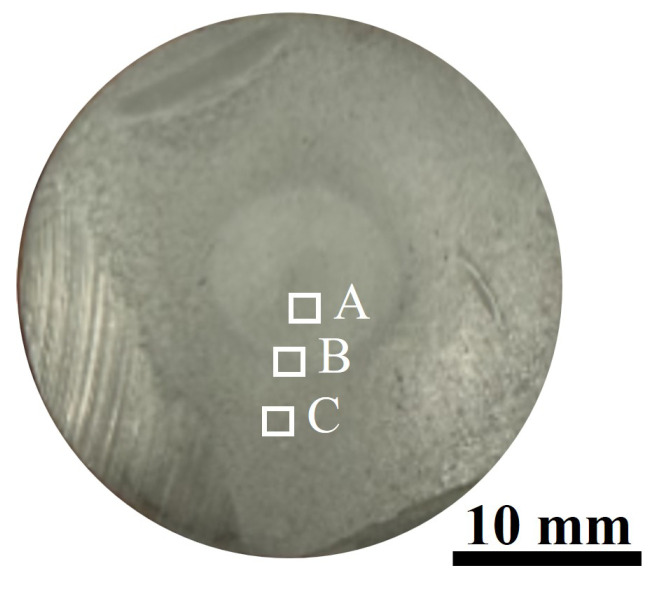
Different areas of the ablated surface of Si${}_{3}$N${}_{4}$/BN fibrous monolithic ceramics after 60 s × 30 of ablation.

To elucidate the ablation behavior of the Si${}_{3}$N${}_{4}$/BN-based whisker structure, we examined the ablation resistance of samples across varying durations. Samples were designated as S1, S2, and S3, based on ablation times of 60 s × 10, 60 s × 20, and 60 s × 30, respectively. Figure 5 presents the post-ablation morphologies of these samples. Notably, the oxidized surface layers of S1, S2, and S3 displayed no discernible defects or macroscopic cracks. This absence suggests the samples withstood the ablation without incurring thermal shock damage, underscoring their robust thermal shock resistance. A protective continuous glass phase, formed by Y${}_{2}$O${}_{3}$ and SiO${}_{2}$, shielded the sample interiors from further degradation. This post-ablation surface morphology offers insights into the material’s thermal shock performance. Notably, the ceramic within this continuous glass phase remained intact throughout. The resilience is attributed to a thin glass-ceramic coating that acts as an additional thermal barrier. This coating reduces the thermal tensile stress within the ceramic and localizes it to the surface [26,27]. Post-polishing, the ablated surfaces of the samples were uniformly smooth. Given their brief exposure, S1 and S2 exhibited cooler temperatures on their reverse sides, retaining their pristine state without succumbing to high-temperature thermal fluxes. In contrast, S3, when exposed to an oxyacetylene flame, developed a near-circular ablation pit, attesting to its superior ablation resistance. A closer look at S3’s post-ablation surface reveals three distinct regions: the ablation center, transition zone, and edge zone, each with unique ablation characteristics. These regions correspond to areas A, B, and C in Figure 6.

### 3.2. Surface Phase Composition

**Figure 7:**
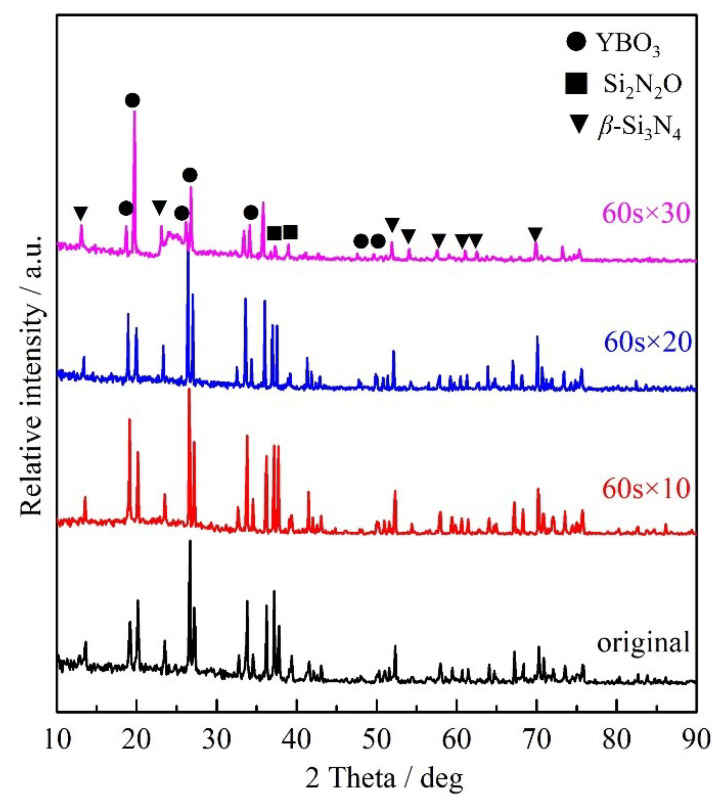
XRD patterns of Si${}_{3}$N${}_{4}$/BN fibrous monolithic ceramics after ablation for different time.

Figure 7 shows the XRD patterns of the ablated samples after different ablation times. For the un-ablated original sample, the relative peak values of β-Si${}_{3}$N${}_{4}$ and BN are higher than those of the ablated samples, indicating that β-Si${}_{3}$N${}_{4}$ and BN underwent thermochemical decomposition during the ablation process. The presence of the oxidation product B${}_{2}$O${}_{3}$ was not detected in 60 s × 10 and 60 s × 20, which forms at high temperatures, has a high evaporation rate, leaving little residue on the surface. Upon subjecting the sample to ablation for 60 s × 30, a continuous glass phase consisting of YBO${}_{3}$ and Si${}_{2}$N${}_{2}$O developed on its surface, safeguarding the sample’s core from additional combustion. Notably, Si${}_{2}$N${}_{2}$O exhibits superior resistance to high-temperature oxidation and demonstrates robust thermal shock resilience, preventing further damage to the internal matrix and ensuring the structural integrity of the ceramic. Studies have found that as the content of Si${}_{2}$N${}_{2}$O increases, the ablation rate decreases, partly due to the increased fracture toughness [28].

### 3.3. Surface Micrographs

**Figure 8:**
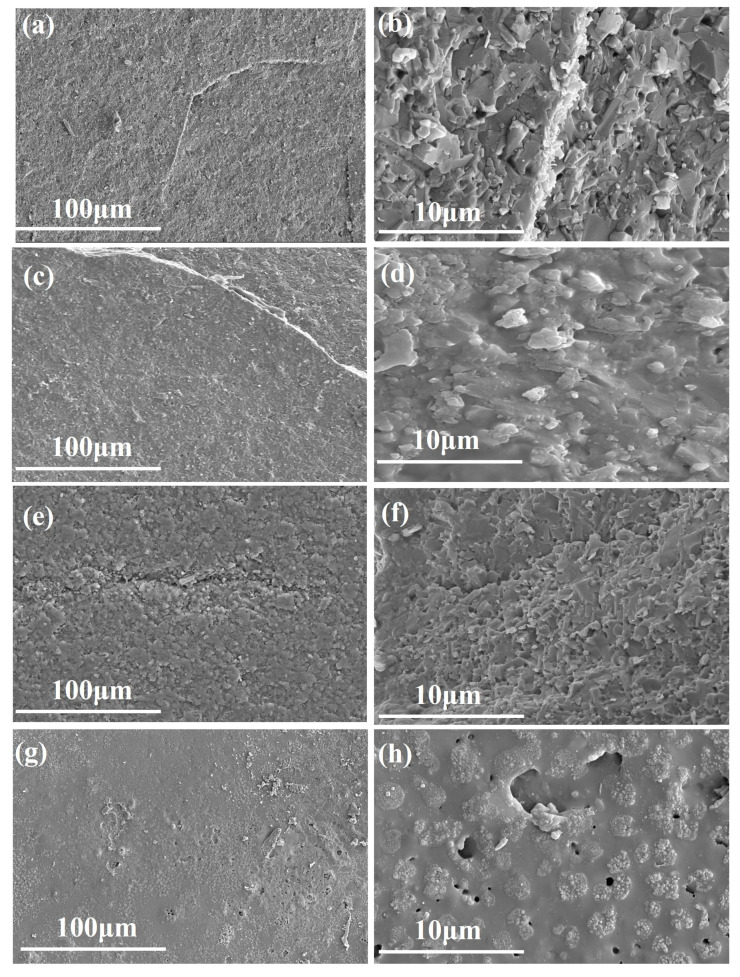
Surface micrographs of the Si${}_{3}$N${}_{4}$/BN fibrous monolithic ceramics after different ablation time. (**a**,**b**) original. (**c**,**d**) 60 s × 10. (**e**,**f**) 60 s × 20. (**g**,**h**) 60 s × 30.

Figure 8 illustrates the surface morphology of Si${}_{3}$N${}_{4}$/BN fibrous monolithic ceramics across various ablation durations. After 60 s × 10 ablation, the surface manifested micro-cracks and micropores, as delineated in Figure 8a,b. This observation can be attributed to two factors: initially, during rapid cooling, significant thermal stresses were imparted onto the ceramics, inducing surface fractures. Subsequently, the oxidation of the ceramics led to the release of B${}_{2}$O${}_{3}$ gas, facilitating micropore formation.

Upon 60 s × 20 ablation, Figure 8c clearly showcases a continuous oxidation layer on the ceramic surface. Concurrently, the surface cracks widened, exhibiting a trend of expansion. The ceramic surface also took on a flake-like configuration. Furthermore, a magnified perspective (Figure 8d) reveals that the dense oxidation layer became more porous when exposed to the acetylene-oxygen flame, thus acquiring a coral-like morphology.

Extending the ablation to 60 s × 30, a surge in the number of cracks was evident, covering a broader expanse of the ceramic surface, as represented in Figure 8e. A detailed examination of the ablated surface (Figure 8f) shows that interparticle gaps on the ceramic surface contracted, leading to a denser oxidation film. However, the presence of micropores remained. Additionally, a pronounced glass phase was identified, punctuated by droplets measuring approximately 50 μm and interspersed with minor voids. As the ablation phase concluded and subsequent cooling began, the residual B${}_{2}$O${}_{3}$ interacted with Y${}_{2}$O${}_{3}$ to produce a borosilicate glass phase. This adherent film remained intact on the material’s surface, thwarting further oxidation by high-speed plasma and thereby significantly bolstering the material’s ablation resistance.

### 3.4. Cross-Sectional Micrographs

**Figure 9:**
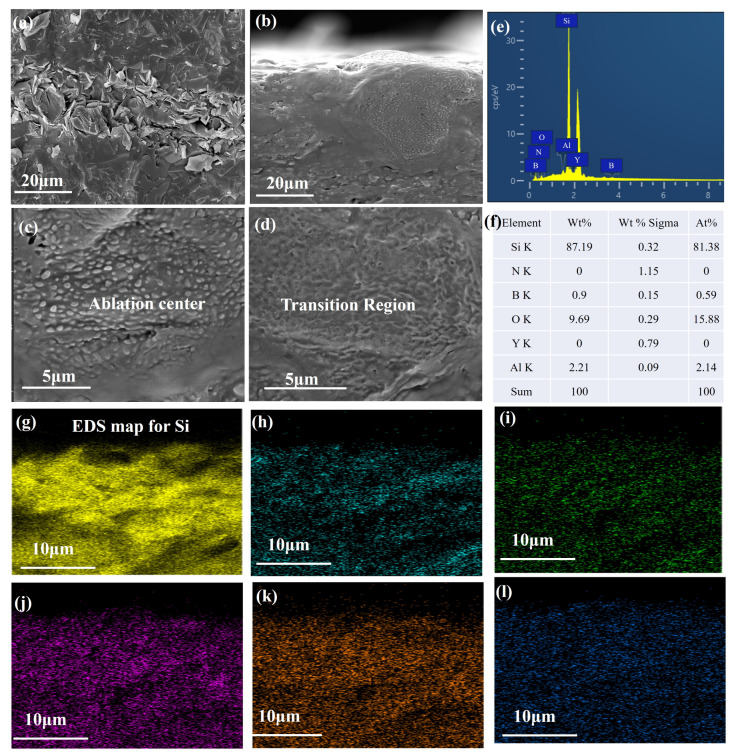
Cross-sectional microstructure, EDS analysis and elemental content of Si${}_{3}$N${}_{4}$/BN fibrous monolithic ceramics before and after 60 s × 30 of ablation. (**a**) original (**b**) low and (**c**,**d**) high magnification of after oxyacetylene ablation, (**e**,**f**) EDS analysis and elemental content, elemental distribution of (**g**) Si, (**h**) N, (**i**) B, (**j**) O, (**k**) Y, (**l**) Al.

To further investigate the structural state of materials post-ablation, the material was dissected and observed. Figure 9 illustrates the microscopic morphology of different cross-sectional areas of the material after 60 s × 30 of ablation. The ablation cross-section, from the material surface to the interior, can be divided into an ablation layer and an original material layer. The total thickness of the ablation layer is approximately 0.2 mm, indicating that the oxyacetylene flame only impacts the material’s surface. The fibrous monolithic ceramic material effectively defends against further internal ablation by the oxyacetylene flame. In the central area, the oxide layer and matrix exhibit good bonding when viewed from the fracture, with minimal pores at the interface. Therefore, the primary ablation mechanisms in the ablation center of this multiphase ceramic composition are thermal oxidation ablation, thermophysical ablation, and mechanical scouring. The ablated surface, from the center to the material edge, can also be divided into three areas: the ablation center area (Figure 9b), transition area (Figure 9c), and edge area.

Figure 9 clearly displays the micrograph of Si${}_{3}$N${}_{4}$/BN fibrous monolithic ceramics after 60 s × 30 of ablation. From the figure, it can be observed that spherical liquid bubbles are formed in both the ablation transition and edge areas. Energy spectrum analysis identifies the composition as SiO${}_{2}$, which assumes a spherical shape under surface tension to minimize surface energy. The oxide layer is well bonded to the matrix, with no pores observed, and has a relatively thick dimension of approximately 4 μm. Energy spectrum analysis of the fracture indicates that the oxide layer in the transition area consists of SiO${}_{2}$, which is flaky and loosely arranged, potentially due to high-speed airflow scouring. Minute pores exist at the interface of the oxide layer and the matrix, indicating moderate bonding. Figure 9d presents the energy spectrum of bubbles in the cross-section after the ceramic material is ablated. Energy spectrum analysis of the liquid bubbles formed on the sample surface post-ablation indicates that the liquid phase bubbles can be considered as SiO${}_{2}$.

### 3.5. Ablation Behavior

Si${}_{3}$N${}_{4}$ decomposes at 1900 °C, BN sublimates at 3000 °C, and SiO${}_{2}$ melts between 1600 and 1700 °C. At an ambient temperature of 2000 °C, Si${}_{3}$N${}_{4}$ partially decomposes and sublimates, with concurrent reactions between BN and O${}_{2}$. During ablation, Si${}_{3}$N${}_{4}$/BN fibrous monolithic ceramics undergo specific chemical reactions.

In an oxygenacetylene environment above 2200 °C, Si${}_{3}$N${}_{4}$/BN fibrous monolithic ceramics undergo significant oxidation and mechanical ablation. The ablation behavior of these ceramics varies with exposure duration. In the initial ablation phase (60 s × 20) at 2000 °C with an oxyacetylene flame, β-Si${}_{3}$N${}_{4}$ decomposes to form liquid SiO${}_{2}$. Influenced by high-speed airflow, this liquid exhibits greater fluidity. The protective glass phase, formed from Si${}_{3}$N${}_{4}$ decomposition and sintering agents, shields the transition zone, preventing further ceramic degradation by high-temperature flames. This zone undergoes thermochemical ablation, thermophysical ablation, and mechanical erosion. At elevated temperatures, acetylene decomposes, producing hydrogen gas and free carbon particles.

During an extended ablation period of 60 s × 30, the amorphous borosilicate glass phase decomposes. Y${}_{2}$O${}_{3}$ and SiO${}_{2}$ combine to form a continuous glass layer on the sample surface. This glass layer, present during ablation, serves as a shield against high-speed flame currents, improving the ablation resistance of Si${}_{3}$N${}_{4}$/BN fibrous monolithic ceramics.

## 4. Conclusions

The Si${}_{3}$N${}_{4}$/BN fibrous monolithic ceramics were subjected to oxyacetylene flame ablation, elucidating the mass ablation rate and linear ablation rate under various ablation durations. By integrating observations of surface morphology, cross-sectional microstructure, and composition of the samples post-different ablation times, an analysis of the ablation mechanism of these ceramics was conducted.

1. The mass and linear ablation rates of Si${}_{3}$N${}_{4}$/BN fibrous monolithic ceramics initially increase with prolonged ablation time, then decrease, followed by a subsequent rise. Specifically, for ablation durations of 60 s × 10, 60 s × 20, and 60 s × 30, the corresponding mass ablation rates are $1\times 10^{-5}$ mg/s, $-8.3\times 10^{-6}$ mg/s, $-6.7\times 10^{-7}$ mg/s, respectively; the linear ablation rates are $4.7\times 10^{-5}$μm/s,$-1.2\times 10^{-5}$μm/s and $1.7\times 10^{-6}$μm/s. The ablation resistance of Si${}_{3}$N${}_{4}$/BN fibrous monolithic ceramics is notably superior to other Si${}_{3}$N${}_{4}$-based ceramics.;
2. With the prolongation of ablation time, a dense oxidation layer forms on the surface of Si${}_{3}$N${}_{4}$/BN fibrous monolithic ceramics. However, after 60 s × 30 of ablation, this oxidation layer is eroded by the oxyacetylene flame, revealing a porous, coral-like structure with numerous cracks. A glass phase, primarily formed from sintering aids and covering the Si${}_{3}$N${}_{4}$ ceramic surface, is observed on the ablated surface of the sample, effectively preventing further ablation.

## Figures and Tables

**Table 1:** Parameters of the raw powders adopted in the research.

Raw Material	Purity (%)	Particle Size (μm)	Manufacturer
α-Si${}_{3}$N${}_{4}$	99	0.5	Hefei Aijia New Material Technology Co., Ltd., Hefei, China
BN	99	1.31	Dandong Chemical Research Institute Co., Ltd., Dandong, China
Y${}_{2}$O${}_{3}$	99.99	2	Shanghai Aladdin Biochemical Technology Co., Ltd., Shanghai, China
Al${}_{2}$O${}_{3}$	98	0.73	Showa Denko, Yokohama, Japan

**Table 2:** The compositional components of the Si${}_{3}$N${}_{4}$/BN fibrous ceramics.

α-Si${}_{3}$N${}_{4}$ (wt.%)	Y${}_{2}$O${}_{3}$ (wt.%)	Al${}_{2}$O${}_{3}$ (wt.%)	Deionized Water (wt.%)	(C${}_{6}$H${}_{7}$N${}_{a}$O${}_{6}$)${}_{x}$ (wt.%)	BN
47.5	1.78	0.72	48.75	1.25	3.0

**Table 3:** Working parameters of oxyacetylene flame.

Parameters	Unit	Numerical Value
Heat flux	Kw/m${}^{2}$	4168.8 ± 418.7
Oxygen flow	L/h	1443
Acetylene flow	L/h	927
Oxygen pressure	MPa	0.4
Acetylene pressure	MPa	0.095
Distance from initial specimen surface to flame nozzle	mm	10
Flame nozzle diameter	mm	2
Flame ablation Angle	${}^{\circ }$	90

**Table 4:** Ablation properties of Si${}_{3}$N${}_{4}$-based ceramics.

Samples	Liner Ablation Rate Rm (μm/s)	Mass Ablation Rate Rd (mg/s)
Si${}_{3}$N${}_{4f}$/BN [22]	$1.3\times 10^{2}$	65
Si${}_{3}$N${}_{4}$-Si${}_{2}$N${}_{2}$O-BN [23]	0.5	$6\times 10^{-4}$
C${}_{f}$/Si${}_{3}$N${}_{4}$ [25]	$1.1\times 10^{2}$	10.7
Si${}_{3}$N${}_{4}$/BN fibrous ceramics	$4.7\times 10^{-5}$	$1.0\times 10^{-5}$
Si${}_{3}$N${}_{4}$/BN fibrous ceramics	$-1.2\times 10^{-5}$	$-8.3\times 10^{-6}$
Si${}_{3}$N${}_{4}$/BN fibrous ceramics	$1.7\times 10^{-6}$	$-6.7\times 10^{-7}$

## Data Availability

Not applicable.
